# Isolation and characterization of the fall Chinook aquareovirus

**DOI:** 10.1186/s12985-017-0839-9

**Published:** 2017-09-05

**Authors:** Negar Makhsous, Nicole L. Jensen, Katherine H. Haman, William N. Batts, Keith R. Jerome, James R. Winton, Alexander L. Greninger

**Affiliations:** 1 Department of Laboratory Medicine, University of Washington, 1616 Eastlake Avenue East, Suite 320, Seattle, WA 98102 USA; 2 Fred Hutchinson Cancer Research Institute, Seattle, WA USA; 3 Washington State Department of Fish and Wildlife, Olympia, WA USA; 4 USGS Western Fisheries Research Center, Seattle, WA USA

**Keywords:** Aquareovirus, Aquareovirus B, Chinook, Fall chinook, Salmon virus

## Abstract

**Background:**

Salmon are paramount to the economy, ecology, and history of the Pacific Northwest. Viruses constitute one of the major threats to salmon health and well-being, with more than twenty known virus species that infect salmon. Here, we describe the isolation and characterization of the fall Chinook aquareovirus, a divergent member of the species *Aquareovirus B* within the family *Reoviridae*.

**Methods:**

The virus was first found in 2014 as part of a routine adult broodstock screening program in which kidney and spleen tissue samples from healthy-appearing, adult fall Chinook salmon (*Oncorhynchus tshawytscha*) returning to a hatchery in Washington State produced cytopathic effects when inoculated onto a Chinook salmon embryo cell line (CHSE-214). The virus was not able to be confirmed by an RT-PCR assay using existing aquareovirus pan-species primers, and instead was identified by metagenomic next-generation sequencing. Metagenomic next-generation sequencing was used to recover the full genome and completed using 3′ RACE.

**Results:**

The genome of the fall Chinook aquareovirus contains 11 segments of double-stranded RNA totaling 23.3 kb, with each segment flanked by the canonical sequence termini found in the aquareoviruses. Sequence comparisons and a phylogenetic analysis revealed a nucleotide identity of 63.2% in the VP7 gene with the Green River Chinook virus, placing the new isolate in the species *Aquareovirus B*. A qRT-PCR assay was developed targeting the VP2, which showed rapid growth of the isolate during the initial 5 days in culture using CHSE-214 cells.

**Conclusions:**

This sequence represents the first complete genome of an Aquareovirus B species. Future studies will be required to understand the potential pathogenicity and epidemiology of the fall Chinook aquareovirus.

## Background

Members of the family *Reoviridae* include viruses with genomes having between 9 to 12 segments of double-stranded RNA [1]. Reoviruses infect a variety of hosts from mammals to fungi and have a wide geographic distribution [1]. The family *Reoviridae* is composed of two sub families: *Spinareovirinae* and *Sedoreovirinae* with 15 different genera between them [2]. Members of the genus *Aquareovirus* have been isolated from a wide variety of aquatic animal hosts in which they produce infections that range from benign to severe hemorrhagic disease [3–5].

Among the seven established species of aquareoviruses (A-G), isolates representing the species Aquareovirus A and B are known to infect a wide variety of salmonids in the Pacific Northwest. They are typically isolated at low prevalence and intensity from populations of returning adult salmonids in which they produce no identified pathology. However, experimental infections of several species of juvenile salmonids have demonstrated Aquareovirus A causes a focal necrotizing hepatitis [6]. In other studies, infections of trout with Aquareovirus A were shown to increase resistance to the highly pathogenic fish rhabdovirus, infectious hematopoietic necrosis virus (IHNV), presumably by the induction of interferon [7].

While aquareoviruses generally grow well in cell culture producing a characteristic plaque-like or syncytial type of cytopathic effect (CPE), their identification is often performed by molecular means such as RT-PCR. The reliance on molecular detection necessitates the availability of complete genome sequences to improve PCR primer design and diagnostic test accuracy and to understand virus diversity and their molecular evolution. Aquareoviruses all contain 11 dsRNA segments that encode for seven structural proteins (VP1-VP7) and 5 nonstructural proteins [2, 8]. While their distribution among the seven aquareovirus species is uneven, approximately 15 aquareovirus genomes have been completed and several strains have been partially completed [2].

Here, we describe the isolation, characterization, and complete genome sequencing of the fall Chinook aquareovirus, a new member of species Aquareovirus B detected in routine surveillance of salmonids. The virus grew in CHSE-214 cell cultures, but failed to be confirmed by diagnostic RT-PCR assays primed with sets of degenerate Aquareovirus pan-species primers. Our results show the genome sequence of the fall Chinook aquareovirus to be the most divergent member of an existing aquareovirus species and will aid in the redesign of diagnostic molecular assays for salmonid aquareoviruses.

## Methods

### Cell culture and Aquareovirus RT-PCR

Tissue samples from adult fall Chinook salmon (*Onchorynchus tshawytscha*) were collected from fish returning to the Washington Department of Fish and Wildlife (WDFW) Glenwood Springs Hatchery in Washington State in October 2014. Five fish pools of kidney and spleen were homogenized and diluted 1:10 by weight in minimum essential medium (MEM). Antibiotic incubation media consisting of 1000 μg/mL penicillin-streptomycin, 50 μg/mL Fungizone and 0.5 μg/mL gentamicin in MEM was used to prepare a 1:4 dilution of the sample and incubated 2 h at 15 °C. A 24- well tissue culture plate was seeded with Chinook salmon embryo (CHSE-214) cells 24 h prior and allowed to grow until a monolayer of >90% confluency was reached. CHSE-214 cells were inoculated with 200 μL per well of sample in duplicate. The inoculum was kept on cells for 1 h at 15 °C before cells were overlayed with MEM containing 5% fetal bovine serum (MEM-5), 0.5 μg/mL gentamycin, 0.5 μg/mL fungizone and 100 μg/mL penicillin-streptomycin and incubated at 15 °C. For 28 days, cells were observed for CPE 2–3 times per week.

RNA from the tissue culture supernatants were extracted with Trizol according to the RNA extraction miniprep protocol (DirectZol, Zymo Research) and screened using sets of degenerate aquareovirus pan-species RT-PCR primers (Table 1)*.* For each test, known positive control RNA from isolates of Aquareoviruses A and B were included along with deioinzed water as a negative control. Forward and reverse primers as shown in Table 1 for either Aquareovirus A (1811F/1812R) or Aquareovirus B (1807F/1809R) were tested in separate reactions. A nested PCR without the reverse transcription step, reverse transcriptase or RNasin enzymes was conducted using internal primers (Table 1) for Aquareovirus A (1813F/1814R) or for Aquareovirus B (1808F/1810R). Because the CPE produced by the fall Chinook aquareovirus on the CHSE-214 cells appeared somewhat different from that in a companion flask of cells infected with an Aquareovirus B isolate from a different location, we performed another RNA extraction and RT-PCRs with primers 1807F/1809R and 1811F/1812R. Additionally, after 13 days post infection, a further RNA extraction and PCR run was tested in combination with several isolates of putative aquareoviruses from locations near the Glenwood Springs Hatchery. The RT-PCR was performed in 50uL reactions using 28uL deionized water, 10uL 5X PCR buffer, 3uL 25 mM MgCl2, 1uL 10 mM dNTP, 1uL of each 20uM each primer, 0.25uL GoTaq, and 0.5uL AMV reverse transcriptase, and 0.25uL RNasin with 5uL of RNA template. RT-PCR conditions were 50C 30 min; 95C 2 min; 30 cycles of 95C 30s, 50C 30s, 72C 1 min; followed by 72C for 7 min and a 15C hold. PCR products were visualized on an ethidium bromide stained 1.5% agarose gel.

**Table 1:** Pan-specific degenerate RT-PCR primers used for (A) identification of isolates of Aquareovirus species A or B in an attempt to identify the fall Chinook aquareovirus (Batts, unpublished data). (B) Modified primers for identification of isolates of species Aquareovirus B based upon sequence analysis that includes the fall Chinook aquareovirus

A.	
Aquareovirus A	
1811F	C RCC ATG GAG ACC AAA CC
1812R	CT STG RTT CAT CAT AGC GTG
1813F	AY ATC ACY CAY SAG TGT CA
1814R	GC TAG ATC TTT GCC ATA GAA
Aquareovirus B	
1807F	C GCC ATG GAT ACC AAG CC
1809R	TG GGA CAG CAG GGC GTG
1808F	AY WWC MCT CAC GAG TGT CA
1810R	GM KAG ATC CTT GCC GTA RAA
B.	
4370F	ACC ATC GCY ATG GAT AYY AAG CC
4371R	CG GTT SGA SCC ATG ACG RTT CTC
4372F	ACC GTW GCC AAC GCA CTT TGC GA
4373R	G CGC GAT TGS WSS TAG AGA GGA

### Library preparation and next generation sequencing

For genome recovery, RNA from supernatant was extracted using the Zymo Viral RNA Kit (Zymo Research) and treated with Turbo DNAse I (Thermo Fisher) [9]. cDNA synthesis was performed using random hexamers with SuperScript III reverse transcriptase and second-strand synthesis was performed using Sequenase v2.0 (Thermo Fisher). Double-stranded DNA was cleaned using a Zymo DNA-5 Clean and Concentrator column (Zymo Research) and library preparation was performed using 0.4X volumes of Nextera XT (Illumina), 16 cycles of dual-indexed library amplification, purification with 0.75X Ampure XP beads (Beckman Coulter). The library was quantified on Qubit 3.0 and Agilent Bioanalyzer 2100 and sequenced using a portion of a 1x180bp sequencing run on an Illumina MiSeq [10].

Sequencing reads were adapter and quality trimmed using cutadapt, repaired using pairfq, and de novo assembled using SPAdes v3.8 [11]. The resultant contigs were aligned by megablast to the NT database and the unaligned contigs were aligned by DIAMOND to the NR database [12]. The alignment files were taxonomically assigned and visualized using MEGAN 2.0, while contigs were visualized in Geneious v9.1 and phylogenies were performed using MAFFT alignments and MrBayes for phylogenetic trees [13–15].

### Genome finishing

Follow-up RT-PCR was performed to close a gap in the NS1 gene using NS1–1386R and NS1-374F primers (Table 2) and quarter-volumes of the Qiagen One-Step RT-PCR kit. 3’RACE was performed as described previously [16]. Briefly, post-DNAsed RNA was polyadenylated by using *E. coli* Poly(A) Polymerase (NEB). Poly(A) RNA was reverse transcribed using Super Script III RT with Q_T_ primer and 30 cycles of Phusion PCR with a T_m_ of 55 °C with the Q _outer_ primer and GSP1 (Table 2) [16]. Nested PCR was performed using 1uL product from the first round PCR with 35 cycles using the Q inner primer and associated GSP2. PCR products were visualized on a 1.5% agarose gel and bands were gel-extracted using the QIAquick Gel Extract kit and sequenced via Sanger sequencing.

**Table 2:** RT-PCR primers used for sequencing the complete genome (A) and for a novel qRT-PCR assay to quantify virus load (B)

A.	
VP1-F	TGT GAC ATC GGC GAC AAC
VP1-R	CTG ACG TGA CCA TGC CAA T
VP2-F	TGG ATG CCC CTA GAC TTT G
VP2-R	TGT CCG AAT TAT GAC AAT AGT C
VP3-F	CCG ATG GTC AGC TCT CG
VP3-F-outer	CAC CCA GAA CAA CAA TGG
VP3-R	GGG GAC TTT GTC TCC GA
VP4-F	TAC TTC CCG TGA CGT CC
VP4-R	TTG AAG CTT GAA CCA AGC GG
VP5-F	CCG CAA TTG GTA CCA CTG
VP5-R	CAG CGG TTC CCA TGA GT
VP6-F	GCG ATG GAA CTC GCT C
VP6-R	GCA TTA AGA TGG GCA CCG
NS2-F	TGA TGT GGC AGA AGC CTG
NS2-R	TGG CGC CAT TGA GAC C
VP7-F	TCT ACG AGC AAT CGC GC
VP7-R	ATA CCT GGT ACT GTC CAG
NS3-F	GTT AAA TGG TAC GGC GGC
NS3-R	TAT GGG TCT GCA GCG TG
NS4-F	GCC TGG TTC GAC TAC ATC
NS4-R	GCA CGT CCA ACC TTC TTG
NS1–2-F	TAC GGC CAA GGT CAG TTC
NS1–2-R	GGT TGG GAA TGA AGC GAA GA
NS1–1-F	CAC AGC TCG GCG TCA C
NS1–1-R	TAA GTG ATT GTC CAG GTG G
B.	
qVP2-F	GGC GTA ATC CAG CCG C
qVP2-R	GCT AGT GAA GGG ATC GTC

### Development of a qRT-PCR assay for assessing virus growth kinetics

After the appearance of initial CPE in the primary culture, three, 25 cm^2^ flasks were seeded with CHSE 214 cells and allowed to incubate at 15 °C overnight with MEM containing 10% fetal bovine serum (MEM-10) until cells were 90–100% confluent. When the cell monolayer was confluent, growth medium was removed and 200 μL of primary culture was filtered through a 0.2 μM membrane into two of the three flasks. The negative control was 200 μL of MEM-10 into a third flask. Flasks were gently swirled and allowed to incubate for 1 h at 15 °C. After 1 h, 7 mL of MEM-5 was added to each flask. A 200uL aliquot for PCR was taken to quantify the starting inoculum. On days 1–11, flasks were observed for CPE and 200 uL aliquots were collected from each flask and frozen at −20 °C.

A quantitative PCR was designed based on alignment of the nucleotide sequences of selected segments of other aquareoviruses. Primers to conserved regions of the viral polymerase (VP2) were designed (Table 2) and a positive control amplicon for use in developing a standard curve was created using the Qiagen One-Step RT-PCR kit.

Double-stranded viral RNAs were extracted from culture supernatant using the Pure Link RNA mini Kit (Thermo Fisher) and eluted with 60uL RNAse free water then frozen at −20 °C until used. Reverse transcription was completed using SuperScript II (Thermo Fisher) following manufacturer’s instructions. cDNA was frozen at −20 °C until qPCR could be completed. Primers provided were optimized using the SYBR Green recommendation for a final primer concentration of 0.3uM. The control standard was quantified using a Qubit (Thermo Fisher) and a known stock solution prepared (10^6^ copies/uL) for use in creating a standard curve.

PCR master mix consisted of a 1X final concentration SYBR Green Master mix (Applied Biosystems), 0.3uM of the forward and reverse primer and 1uL of cDNA template for a final volume of 10uL/ rxn. Samples were run in triplicate using a 384 well plate in a Quant studio 6 Flex PCR machine (Applied Biosystems). The standard curve was prepared as a dilution series of 10^6^ to 10^1^ copies/rxn.

## Results

### Isolation of the fall Chinook aquareovirus

**Fig. 1:**
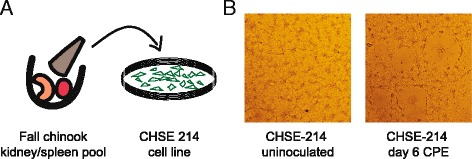
Characteristics of the fall Chinook aquareovirus. **a** Virus was recovered from kidney-spleen homogenates based on cytopathic effect on CHSE-214 cells. **b** Syncytia formation characteristic of Aquareovirus was visible as early as day 4 on CHSE-214. Plaques from day 6 on inoculated CHSE-214 cells are shown compared with uninoculated CHSE-214 cells

**Fig. 2:**
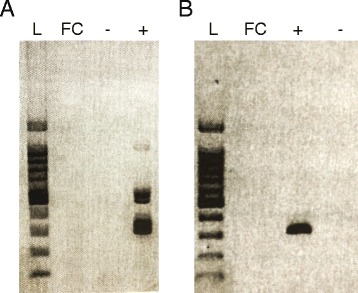
Agarose gels showing results of RT-PCR assays for the fall Chinook aquareovirus. **a** RT-PCR assays for Aquareovirus A: lane 1, 100 bp molecular weight marker; lane 2, fall Chinook aquareovirus; lane 3, negative control; lane 4, positive control. **b** RT-PCR assays for Aquareovirus B: lane 1, 100 bp molecular weight marker; lane 2, fall Chinook aquareovirus; lane 3, positive control; lane 4, negative control

During the initial incubation of samples at the WDFW laboratory, CPE appeared on day 28 in only 1 of 2 wells of CHSE-214 cells inoculated with 1 of the 13 pools of kidney/spleen samples (Fig. 1a), indicating the virus was present in the population at low prevalence and intensity of infection. Beginning on day 4, flasks of CHSE-214 cells inoculated with filtered cell culture fluid from the affected well developed CPE typical of most aquareoviruses consisting of plaque-like areas of syncytia (Fig. 1b). Viral stocks were prepared by filtering culture fluid into cryovials that were frozen at −80 °C and sent to the United States Geological Survey Western Fisheries Research Center for testing. RT-PCR assays using several pairs of aquareovirus pan-species primers failed to amplify the extracted RNA (Fig. 2). Subsequently, the unidentified isolate was submitted to the Department of Laboratory Medicine at the University of Washington for next-generation sequencing.

### Genome characterization of Fall Chinook reovirus

A total of 187,569 adapter-trimmed reads were recovered from the RNA-sequencing libraries from the original cell culture supernatant. De novo assembly of the RNA-sequencing reads yielded 42 contigs longer than 500 bp. BLASTN and DIAMOND alignment indicated the presence of a novel aquareovirus, as alignments to various aquareoviruses over all eleven segments were found among the top 100 contigs.

**Fig. 3:**
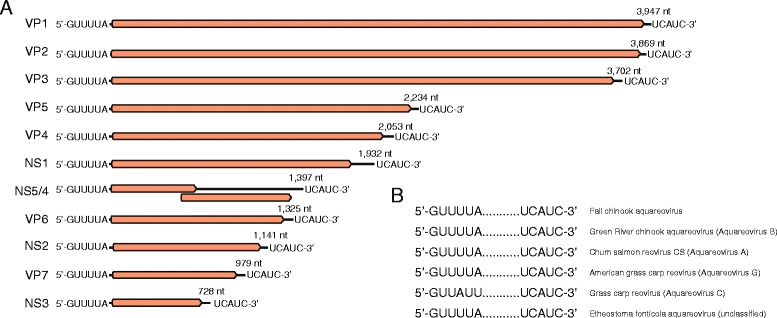
Fall Chinook aquareovirus genome organization. **a** Metagenomic RNA sequencing of supernatants and 3’RACE genome finishing yielded a novel aquareovirus with 11 complete dsRNA segments. **b** 5′ and 3′ ends of the virus revealed canonical repeats as found in other aquareovirus B species

Because of the dsRNA nature of the reovirus genome, 3’RACE was performed to finish both 5′ and 3′ ends. The fall Chinook aquareovirus genome contained 11 segments totaling 23.3 kb, ranging in size from 3947 bp to 728 bp (Fig. 3a). 5′NTR length ranged from 11 to 30 bp while the 3′NTR length ranged from 30 to 157 bp. All segments began and ended with the canonical aquareovirus sequence (5′-GUUUA and UCAUC-3′, Fig. 3b). All segments contained a single ORF, except S7 which contained two overlapping ORFs, suggesting that the fall Chinook aquareovirus encodes for 12 proteins. In total, 39,633 of the 187,569 adapter-trimmed reads (21.1%) mapped to the finished fall Chinook aquareovirus genome.

### Phylogenetic analysis of fall Chinook aquareovirus

**Fig. 4:**
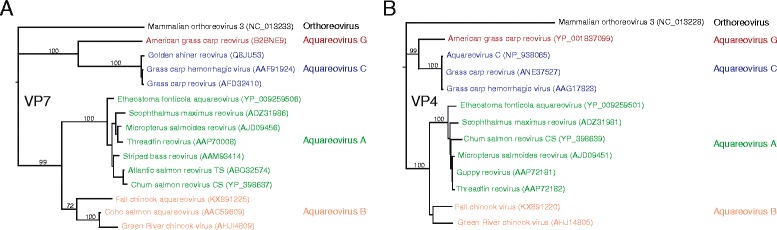
Phylogenetic analysis of the fall Chinook aquareovirus. Fall Chinook aquareovirus is a member of the aquareovirus B species based on sequence alignment and phylogenetic analysis of the VP7 (**a**) and VP4 (**b**) segments

The fall Chinook aquareovirus VP7 shares a 63% nucleotide identity with the Green River Chinook virus (KC588384) and 47% amino acid identity with the Coho salmon aquareovirus (AAC59609). Phylogenetic analysis of both the VP4 and VP7 segments revealed clustering of the fall Chinook aquareovirus within the species Aquareovirus B (Fig. 4). Of note, the fall Chinook aquareovirus demonstrates the most divergence of any new aquareovirus that does not constitute a new species based on the ICTV definition as well as the first complete genome sequence for an aquareovirus B species. Examination of the VP2 coding sequence revealed the canonical amino acid motifs for polymerase (DXXXXD, SG, and GDD) present in members of the *Aquareovirus* and *Orthoreovirus* genera.

Pairwise nucleotide identity between the Green River chinook aquareovirus and the fall Chinook aquareovirus ranged from 63% (VP7) to 73% (VP3). The existing pan-Aquareovirus B primers had 3 mismatches in each of the outer primers and 9 mismatches in the hemi-nested second round primer in the VP7 segment, consistent with their inability to amplify the fall Chinook aquareovirus, and leading to the design of a novel primer set for confirmation of isolates of species Aquareovirus B (Table 1).

### Virus growth kinetics of fall Chinook aquareovirus

**Fig. 5:**
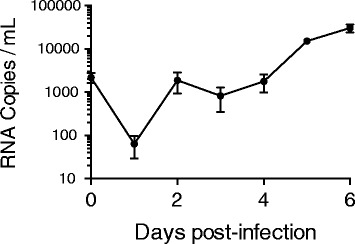
Serial qRT-PCR on targeting VP2 segment demonstrated viral RNA levels increased in culture to day 6 consistent with viral growth on CHSE-214 cells

Recovery of the full genome sequence of the fall Chinook aquareovirus allowed development of a qRT-PCR assay that was used to study viral growth kinetics in flasks containing monolayer cultures of CHSE-214 cells. Samples of culture fluid removed from the flasks at daily intervals showed a strong increase in virus titer during the initial incubation period (Fig. 5).

## Discussion

We describe the identification, culture, and complete genome sequencing of a novel aquareovirus in fall Chinook salmon from Washington State. We have named this new virus the fall Chinook aquareovirus. Like all members of the *Aquareovirus* genus, the fall Chinook aquareovirus contains 11 dsRNA segments. Its culture in CHSE 214 cells was associated with the canonical large plaque-like syncytia CPE. The ICTV defines a new aquareovirus species based a variety criteria, including sequence alignment of the VP7 segment as having less than 55% nucleotide sequence identity and less than 36% amino acid sequence identity [17]. The fall Chinook aquareovirus thus constitutes a novel member of the species Aquareovirus B.

The virus was recovered from kidney-spleen pools of asymptomatic fall Chinook salmon in Washington State. Although they commonly cause cytopathic effect in cell culture, aquareoviruses of Pacific salmon are typically found in low prevalence among returning adult fish without illness and it is unclear what, if any, loss of function or sequelae they are associated.

The discovery and characterization of this virus will allow for the development of improved diagnostic markers as the existing aquareovirus pan-species molecular assays failed to detect this viral genome. Given that aquareoviruses grow well in culture, whole genome sequencing of aquareovirus genomes can be accomplished at low cost with relatively few sequencing reads. More whole genome sequences of aquareoviruses are needed to ensure diagnostic integrity and to understand the basis of molecular evolution in this genus. Indeed, currently there are sequence data available for only three members from species Aquareovirus B in NCBI Genbank of which the fall Chinook aquareovirus represents the first complete genome sequence. Only a partial sequence of segment 10, encoding VP7 is available for the Coho salmon aquareovirus (U90430.1) and the Green River chinook virus is missing segment four and approximately 3 kb from the VP2 sequence of segment two (KC588377.1).

This virus also illustrates the importance of routine surveillance for discovering new fish viruses that may not be associated with pathogenicity. The added sequence diversity here will inform the molecular mechanism by which aquareovirus induce interferon and why certain species are more pathogenic than others. Interferon induction by aquareoviruses has shown to be a potential protective mechanism against other more pathogenic viruses such as IHNV [7]. Future studies will also be required to understand the geographical and temporal breadth and evolution of fall Chinook aquareovirus, and whether it has pathogenicity in other fish species [8].

## Conclusion

We describe the isolation, genome sequencing, and characterization of the fall Chinook aquareovirus, a novel aquareovirus B member, during routine surveillance of salmon in Washington State. This virus failed to be amplified by exisiting aquareovirus B RT-PCR primers and represents the first complete genome sequence of an aquareovirus B.

## Abbreviations

CPE: Cytopathic effect
dsRNA: Double-stranded ribonucleic acid
MEM: Minimum essential medium
RT-PCR: Reverse transcription polymerase chain reaction
WDFW: Washington Department of Fish and Wildlife

## References

[1] Dermody TS (1998). Molecular mechanisms of persistent infection by reovirus. Curr Top Microbiol Immunol.

[2] Zhang Q, Gui J-F (2015). Virus genomes and virus-host interactions in aquaculture animals. Sci China Life Sci.

[3] Crane M, Hyatt A (2011). Viruses of fish: an overview of significant pathogens. Viruses.

[4] Subramanian K, McPhillips TH, Samal SK (1994). Characterization of the polypeptides and determination of genome coding assignments of an aquareovirus. Virology.

[5] Kibenge FSB, Godoy MG (2016). Aquaculture virology. Reoviruses of aquatic organisms.

[6] Winton JR, Lannan C., Yoshimizu M, Kimura T. Response of Salmonid Fish to Artificial Infection with Chum Salmon Virus, p. 270–278. In Viruses of Lower Vertebrates. Berlin: Springer-Verlag; 1987.

[7] LaPatra SE, Lauda KA, Jones GR (1995). Aquareovirus interference mediated resistance to infectious hematopoietic necrosis virus. Vet Res.

[8] Chen Z-Y, Gao X-C, Zhang Q-Y (2015). Whole-genome analysis of a novel fish reovirus (MsReV) discloses aquareovirus genomic structure relationship with host in saline environments. Viruses.

[9] Greninger AL, Chen EC, Sittler T, Scheinerman A, Roubinian N, Yu G, Kim E, Pillai DR, Guyard C, Mazzulli T, Isa P, Arias CF, Hackett J, Schochetman G, Miller S, Tang P, Chiu CY (2010). A metagenomic analysis of pandemic influenza A (2009 H1N1) infection in patients from North America. PLoS One.

[10] Greninger AL, Zerr DM, Qin X, Adler AL, Sampoleo R, Kuypers JM, Englund JA, Jerome KR (2017). Rapid metagenomic next-generation sequencing during an investigation of hospital-acquired human parainfluenza virus 3 infections. J Clin Microbiol.

[11] Bankevich A, Nurk S, Antipov D, Gurevich AA, Dvorkin M, Kulikov AS, Lesin VM, Nikolenko SI, Pham S, Prjibelski AD, Pyshkin AV, Sirotkin AV, Vyahhi N, Tesler G, Alekseyev MA, Pevzner PA (2012). SPAdes: a new genome assembly algorithm and its applications to single-cell sequencing. J Comput Biol.

[12] Buchfink B, Xie C, Huson DH (2015). Fast and sensitive protein alignment using DIAMOND. Nat Methods.

[13] Huson DH, Beier S, Flade I, Górska A, El-Hadidi M, Mitra S, Ruscheweyh H-J, Tappu R (2016). MEGAN Community Edition - Interactive exploration and analysis of large-scale microbiome sequencing data. PLoS Comput Biol.

[14] Katoh K, Misawa K, Kuma K, Miyata T (2002). MAFFT: a novel method for rapid multiple sequence alignment based on fast Fourier transform. Nucleic Acids Res.

[15] Huelsenbeck JP, Ronquist F (2001). MRBAYES: Bayesian inference of phylogenetic trees. Bioinforma Oxf Engl.

[16] Scotto-Lavino E, Du G, Frohman MA (2007). 3′ End cDNA amplification using classic RACE. Nat Protoc.

[17] Mohd Jaafar F, Goodwin AE, Belhouchet M, Merry G, Fang Q, Cantaloube J-F, Biagini P, de Micco P, Mertens PPC, Attoui H (2008). Complete characterisation of the American grass carp reovirus genome (genus Aquareovirus: family Reoviridae) reveals an evolutionary link between aquareoviruses and coltiviruses. Virology.

